# A Four-Monoclonal Antibody Combination Potently Neutralizes Multiple Botulinum Neurotoxin Serotypes C and D

**DOI:** 10.3390/toxins13090641

**Published:** 2021-09-10

**Authors:** Consuelo Garcia-Rodriguez, Shude Yan, Isin N. Geren, Kristeene A. Knopp, Jianbo Dong, Zhengda Sun, Jianlong Lou, Fraser Conrad, Wei-Hua Wen, Shauna Farr-Jones, Theresa J. Smith, Jennifer L. Brown, Janet C. Skerry, Leonard A. Smith, James D. Marks

**Affiliations:** 1 Department of Anesthesia & Perioperative Care, University of California, San Francisco, Zuckerberg San Francisco General Hospital and Trauma Center, 1001 Potrero Avenue, San Francisco, CA 94110, USA; maconsuelo.gr@gmail.com (C.G.-R.); shudeyan@yahoo.com (S.Y.); isin.geren@medeniyet.edu.tr (I.N.G.); Kristeeneknopp@gmail.com (K.A.K.); jianbodong@gmail.com (J.D.); zhengda_sun@hotmail.com (Z.S.); jianlong.lou@ucsf.edu (J.L.); fraser.conrad@ucsf.edu (F.C.); wei.wen@ucsf.edu (W.-H.W.); shauna.farr-jones@ucsf.edu (S.F.-J.); 2 Molecular and Translational Sciences Division, United States Army Medical Institute of Infectious Diseases (USAMRIID), Fort Detrick, MD 21702, USA; terrys2much@comcast.net; 3 Ke’aki Technologies LLC, United States Army Medical Institute of Infectious Diseases (USAMRIID), Fort Detrick, MD 21702, USA; jennifer.l.brown436.ctr@mail.mil (J.L.B.); jcskerry@gmail.com (J.C.S.); 4 Medical Countermeasures Technology, United States Army Medical Institute of Infectious Diseases (USAMRIID), Fort Detrick, MD 21702, USA; leonard.a.smith@comcast.net

**Keywords:** botulinum neurotoxin, oligoclonal antibodies, serotype C botulism, serotype D botulism, recombinant antibodies, antibody engineering, mouse neutralization assay, botulinum antitoxin

## Abstract

Human botulism can be caused by botulinum neurotoxin (BoNT) serotypes A to G. Here, we present an antibody-based antitoxin composed of four human monoclonal antibodies (mAbs) against BoNT/C, BoNT/D, and their mosaic toxins. This work built on our success in generating protective mAbs to BoNT /A, B and E serotypes. We generated mAbs from human immune single-chain Fv (scFv) yeast-display libraries and isolated scFvs with high affinity for BoNT/C, BoNT/CD, BoNT/DC and BoNT/D serotypes. We identified four mAbs that bound non-overlapping epitopes on multiple serotypes and mosaic BoNTs. Three of the mAbs underwent molecular evolution to increase affinity. A four-mAb combination provided high-affinity binding and BoNT neutralization of both serotypes and their mosaic toxins. The mAbs have potential utility as therapeutics and as diagnostics capable of recognizing and neutralizing BoNT/C and BoNT/D serotypes and their mosaic toxins. A derivative of the four-antibody combination (NTM-1634) completed a Phase 1 clinical trial (Snow et al., Antimicrobial Agents and Chemotherapy, 2019) with no drug-related serious adverse events.

## 1. Introduction

Botulism is an acute life-threatening flaccid paralysis caused by botulinum neurotoxins (BoNTs) produced by *Clostridium* species [1,2]. At least seven immunologically distinct serotypes of BoNTs have been reported: A–G [3,4]. The majority of naturally occurring human botulism is caused by serotypes A, B, E, and F. While BoNT C and BoNT/D primarily intoxicate non-humans, there is significant evidence that both serotypes can intoxicate humans. BoNT/CD causes botulism in avian species, both in the wild and in domestic flocks [5,6,7]. Massive outbreaks have occurred in wild waterfowl [8,9,10]. Type C typically occurs in mammals such as dogs [11,12,13], mink [14], horses [15,16], and cattle [17]. BoNT/D and mosaic-type BoNT/DC botulism outbreaks are rare and are usually associated with horses [18] and cattle [19,20,21]. In cattle, outbreaks have high fatality rates [22,23]. Rare human outbreaks of botulism due to types C and D have been reported [24,25,26]; two cases of foodborne botulism and one case of infant botulism were attributed to BoNT/C [27], while several people fell ill from BoNT/D botulism [24] attributed to tainted ham. 

Mosaic-type BoNT/CD was shown to be therapeutically active in treating dystonia in humans [28] and BoNT/D was proposed as an alternative treatment for patients not responding to BoNT/A or BoNT/B drugs [29]. Both BoNT/C and BoNT/DC are lethal to non-human primates when exposed via the aerosol route [30]. 

BoNT serotypes A, B, E, and F all exist as multiple subtypes which differ from each other in amino acid sequence to an extent that impacts the ability of monoclonal antibodies (mAbs) and polyclonal serum to bind and neutralize the toxin [4,31,32,33,34]. No subtypes of BoNT/C or BoNT/D have been reported: published BoNT/C and BoNT/D sequences each show ≥99.9% identity within toxin type/variant [27,35,36,37]. While serological analysis indicated the presence of both C and D serotypes in some clostridial species, subsequent sequence analysis revealed that these are mosaic toxins, i.e., toxins that contain portions of both BoNT/C and BoNT/D as well as a unique sequence [38]. Mosaic BoNT/CD has high identity to BoNT/C for the amino-terminal two thirds of the toxin (light chain (LC) and translocation domain H_N_) and high identity to BoNT/D for the carboxy-terminal one third (binding domain H_C_). Similarly, mosaic BoNT/DC has high identity to the BoNT/D LC (96%) and H_N_ (92%) and significant identity (78%) to BoNT/C H_C_.

The high potency and lethality of BoNTs make them the highest level of biothreat agent [39]. Because of the threat of the use of BoNTs by those of ill intent, the Public Health Emergency Medical Countermeasure Enterprise (PHEMCE) has a requirement for polyclonal BoNT antitoxin for the National Stockpile in the case of intentional botulism [40]. The antitoxin that is currently stockpiled for treatment for adult botulism is derived from horse plasma, and equine botulism antitoxin (BAT) treats botulism due to serotypes A–G [41]. As BAT is a non-human protein, it is immunogenic, and adverse effects including serum sickness and asystole have been reported [41]. To reduce immunogenicity, the equine antibodies are proteolyzed to remove the Fc portion of the equine IgG, resulting in a combination F(ab’) and F(ab’)2 product. However, Fc removal also reduces the antibody’s half-life and as a result, BAT serum half-lives range from 7.5 to 34.2 h, depending on the serotype [41]. Such a short half-life means that BAT cannot be used to prevent botulism. Furthermore, there have been reports of relapses of human botulism after treatment with BAT [42]. The use of BAT in a mass casualty situation could be challenging or unfeasible because BAT requires slow IV infusion in a volume of 200 mL [41]. As an alternative, the National Institute of Allergy and Infectious Diseases (NIAID) has funded next-generation, recombinant antibody antitoxins that are serotype specific. It has previously been shown that monoclonal antibody combinations (three mAbs/serotype) potently neutralize BoNT through several mechanisms, including eliciting first pass clearance through the liver [31,32,43,44]. Single mAbs or mAb pairs do not provide the requisite potency. A combination of three mAbs increases the potency by two to three orders of magnitude compared to individual mAbs [31,43]. 

While vaccines for botulism in cattle due to type C and D toxin [45] are being designed, this is not a priority for humans due to the rarity of these serotypes causing disease. 

For this work, we sought to generate human BoNT/C and BoNT/D mAbs that, when combined, potently neutralized each of these BoNTs and their mosaic toxins. There is a general requirement for no fewer than three mAbs to potently neutralize a particular toxin serotype. This would mean a BoNT/C and BoNT/D combination antitoxin would require six mAbs. Given the high level of sequence homology in BoNT/C, BoNT/D, and their mosaics, we focused on identifying broadly reactive mAbs that could bind and neutralize both BoNTs and their mosaic toxins to decrease the number of mAbs required for effective protection against both toxin types.

## 2. Results

### 2.1. BoNT/C and BoNT/D Sequence Analysis and Modeling

To better understand differences and similarities in the structural epitopes of BoNT/C and BoNT/D, we aligned their amino acid sequences [46] and analyzed their identity by domain (Table 1). While overall BoNT/C and BoNT/D differed from each other by 49%, the H_N_ of the two BoNTs and their mosaics differed at most by 32%. This observation suggested that it might be possible to generate cross-reactive mAbs binding the H_N_ domain of BoNT/C and BoNT/D and their mosaic toxins. This level of difference is similar to the 31.6% difference between BoNT/F1 and BoNT/F7, where we were successful in generating cross-reactive and cross-protective antibodies. [31].

**Figure 1:**
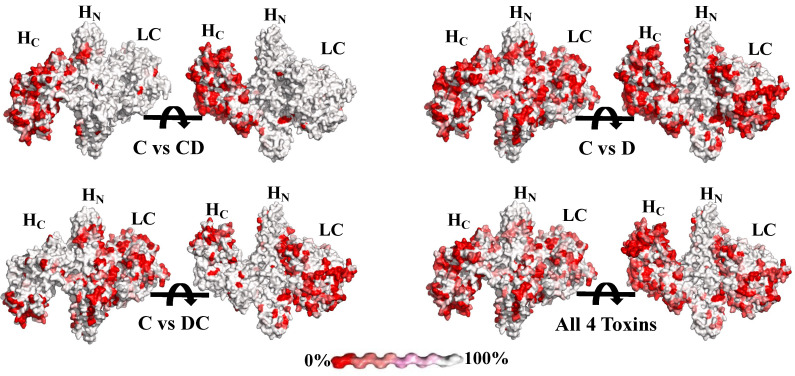
Comparison of sequences of BoNT/C and BoNT/D and their mosaic toxins. The BoNT/C model was constructed by merging the BoNT/C LC crystal structure (pdb ID: 2QN0) and a model of the BoNT/C LC-H_N_ based on the BoNT/A crystal structure (pdb ID: 2NYY). Sequences were as shown in [46]. BoNT/CD, BoNT/DC and BoNT/D were compared to the BoNT/C by using the tool Multalin (http://multalin.toulouse.inra.fr/multalin, accessed on 2 September 2021) and were visualized using ESPript 3 (http://espript.ibcp.fr, accessed on 2 September 2021). The physiochemical similarity of amino acids is indicated on a color scale between red and white. White indicates 100% amino acid identity and red indicates 0% no identity. The panel “All 4 toxins” designates a comparison between identity and differences of all four BoNTs simultaneously.

To visualize epitope differences between BoNT/C and BoNT/D, a structural model of BoNT/C was generated. Side chain differences between BoNT/C and BoNT/CD, BoNT/DC and BoNT/D were then modeled, and the chemical differences of each amino acid side chain were compared, where side chains that are identical are colored white and the most chemically different side chains are colored red (Figure 1). This approach was previously applied to BoNT/A, BoNT/B, BoNT/E, and BoNT/F.

The results of the modeling (Figure 1) identified regions in the H_N_ and LC that are identical or homologous in both BoNT/C and BoNT/D and in the mosaic toxins that could provide targets for the generation of cross-reactive antibodies. This result confirmed our hypothesis that amino acid identities among these toxins should provide ample antigenic targets for the development of cross-reactive mAbs capable of binding and neutralizing these BoNT serotypes and variants. 

### 2.2. Generation and Characterization of Human Monoclonal Antibodies

To generate recombinant mAb-based antitoxins to treat type C and D botulism, previously generated yeast-displayed scFv libraries constructed from humans immunized with pentavalent BoNT toxoid (serotypes A, B, C, D, and E) [32] were sorted for binding to BoNT/C and to BoNT/DC. BoNT/DC was used for sorting, as BoNT/D was not commercially available. BoNT/DC is being sold as BoNT/D, but in fact is from a strain that produces BoNT/DC [47].

A panel of 15 unique scFvs binding BoNT/C were isolated by sorting libraries on pure BoNT/C toxin (Table 2). The mAbs ranged in affinity from 0.19–64 nM, with a mean of 15.6 nM. A panel of 13 scFvs binding BoNT/DC were isolated by sorting libraries on pure BoNT/DC toxin. The mAbs ranged in affinity from 0.5–182 nM, with a mean of 33.6 nM (Table 2). Yeast-displayed scFvs were then screened for binding to crude culture supernatants prepared from Clostridial strains producing BoNT/CD and BoNT/D (Table 2). Since the concentration of BoNT in the supernatants is not known, it was not possible to measure a K_D_ value and only the presence or absence of binding is shown. These studies identified four scFv antibodies, 4C4, 8DC1, 4C10 and 8DC4, that bound to BoNT/C, BoNT/CD, BoNT/DC, and BoNT/D. Two of these cross-reactive scFvs came from sorting on BoNT/C (4C4, 4C10) and two from sorting on BoNT/DC (8DC1, 8DC4). Other mAbs were generated (e.g., 4C2, 87C1, 8DC8) that bound pairs of BoNTs, such as BoNT/C and BoNT/CD, or BoNT/DC and BoNT/D. IgGs were constructed from nine of the scFvs, including the four scFvs binding BoNT/C, BoNT/CD, BoNT/DC, and BoNT/D. As observed with scFvs binding other BoNT serotypes, IgG affinities were generally higher than the scFvs from which they were constructed (Table 2).

### 2.3. Epitope Mapping

**Figure 2:**
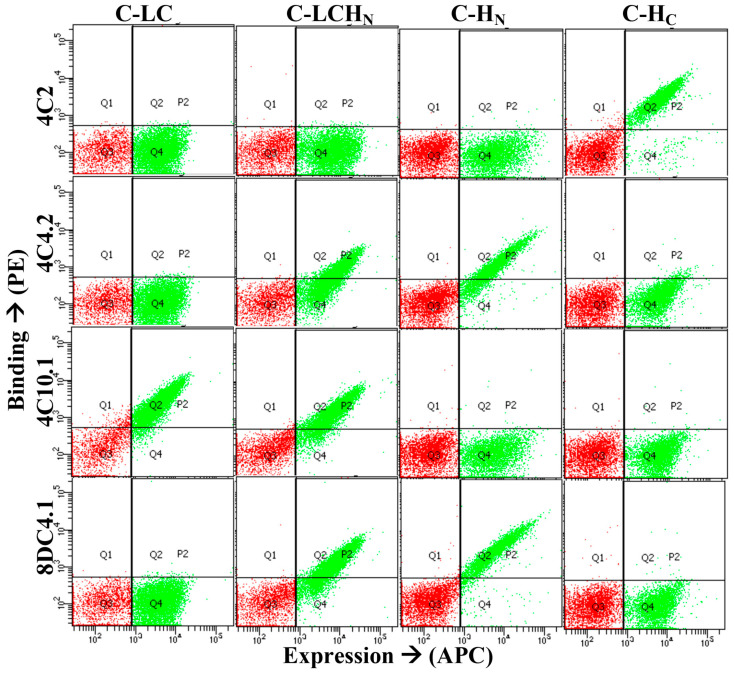
Toxin Domain Binding Assay. Unlabeled IgG 4C2, 4C4.2, 4C10.1 and 8DC4.1 were analyzed for binding to yeast-displaying BoNT/C light chain C-LC), light chain–translocation domain (C-LC-H_N_), translocation domain (C-H_N_) or binding domain (C-H_C_). Bound IgGs were detected using goat anti-human Fc specific, PE-labeled antibody. The yeast display level (expression) for each yeast-displayed domain was quantitated by staining with anti-SV5-AlexaFluor488. Each FACS plot shows independent assays for each IgG on each domain.

**Figure 3:**
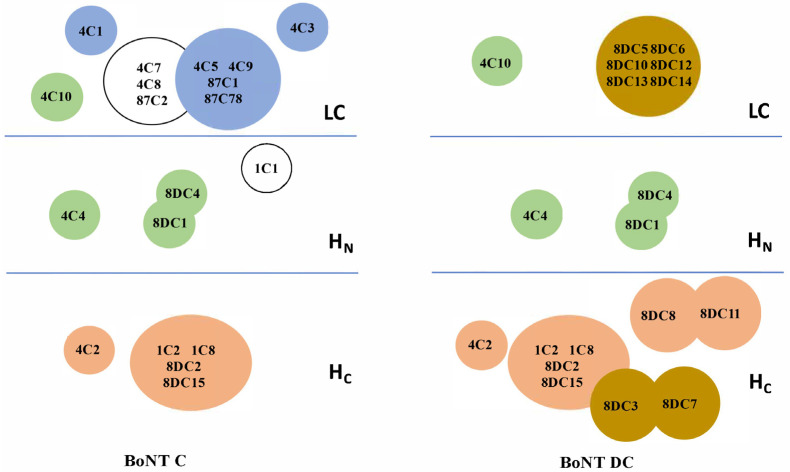
Domain epitope map of BoNT/C and BoNT/DC mAbs. The BoNT domain bound by each mAb was determined using yeast-displayed BoNT/C (**left panel**) or BoNT/DC (**right panel**) domains and either IgG or scFv mAbs, as described in Figure 2. Epitope overlap was determined using a sandwich assay where BoNT/C or BoNT/DC was captured by yeast-displayed scFvs and the ability of each of the other mAbs to simultaneously bind determined using flow cytometry. Epitopes are represented by colored circles with the color indicating the cross reactivity of the mAb(s): white: binds BoNT/C only, blue: Binds BoNT/C and BoNT/CD, peach: Binds BoNT/C and BoNT/DC, green: Binds all four toxins. mAb names within a circle completely overlap and cannot simultaneously bind. Circles that partially overlap indicate that the mAbs interfere with the binding of the other mAb, as indicated by a significantly reduced binding signal.

The BoNT domain bound by each mAb was determined by staining yeast-displayed BoNT/C or BoNT/DC light chains (LCs), the translocation domain (H_N_), the light chain–translocation domain (LC-H_N_), and the binding domain (H_C_) with each antibody, as previously described, using either IgG or purified scFv [48]. Examples of results from these assays are shown in Figure 2 for the IgGs 4C2, 4C4.2, 4C10.1 and 8DC4.1. The latter three IgGs are higher-affinity variants of 4C4, 4C10 and 8DC4 which yielded better binding signals. IgGs 4C2, 4C4.2, 4C10.1 and 8DC4.1 bind the BoNT/C H_C_, H_N_, LC and H_N_, respectively (Figure 2). Epitope overlap was determined by incubating BoNT/C or BoNT/DC with yeast-displayed scFvs and probing the captured BoNT with each of the other scFvs or IgGs, as previously described [31]. Using these two assays, 13 of the 28 mAbs in Table 2 could be mapped to a specific BoNT/C or BoNT/DC domain and a determination could be made as to whether the epitope overlapped with other mAbs. These thirteen mAbs bound seven non-overlapping epitopes: two on the LC, three on the H_N_ and two on the H_C_ (Figure 3). The four mAbs (4C4.2, 4C10.1, 8DC1.2, and 8DC4.1) that recognized all four toxins bound to three non-overlapping epitopes, two on the H_N_ and one on the LC (Figure 3). MAbs 4C4 and 4C10 bound unique epitopes, while 8DC1 and 8DC4 recognized an overlapping epitope. The location of the epitopes of these cross-reactive mAbs on the LC and H_N_ is consistent with the locations of conserved amino acids in the BoNT models shown in Figure 1. A fourth antibody, 4C2, bound BoNT/C and BoNT/DC with high affinity, making it an additional candidate for neutralization studies. Thus, these studies identified four mAbs, binding non-overlapping epitopes in each of the domains shared by BoNT/C, BoNT/CD, BoNT/DC and BoNT/D, that could serve as lead antibodies for a recombinant antitoxin for BoNT/C and BoNT/D.

### 2.4. Affinity Maturation

The affinity of the individual mAbs in an antibody combination impacts potency; the higher the affinity, the great the potency [31,32]. Affinity maturation of each of the cross-reactive antibodies (4C4, 4C10, 8DC1, and 8DC4) was undertaken to improve the affinity, with a goal of a K_D_ less than 1 nM. To increase the potency of the mAb combinations, we affinity matured each of the cross-reactive mAbs 4C4, 4C10, 8DC1, and 8DC4 that bound BoNT/C, BoNT/CD, BoNT/DC, and BoNT/D. Affinity was increased by diversifying the mAb sequence using light chain shuffling, site-directed mutagenesis, or both [49,50,51]. Higher affinity mutants were selected from mutant yeast libraries using FACS. Individual clones were characterized by antibody sequence and scFv affinity. Selected clones were converted to human IgG1 and mAb affinity was measured by using flow fluorimetry (Table 3). Depending on the mAb, this process was iterated one to five times to achieve a K_D_ for each of the BoNT types of less than 1 nM and optimally less than 100 pM. As shown in Table 3, this goal was achieved for each of the four mAbs. Affinities for BoNT/C increased from 400 pM to greater than 1.8 nM to final K_D_ values of 1.1 to 147 pM for BoNT/C and from 950 pM to 600 nM to final K_D_ values of 15 to 87 pM for BoNT/DC, with similar affinity improvements seen for BoNT/CD and BoNT/D. In all cases except for one, affinities were increased using affinity maturation, with overall affinity increases ranging from 2.3-fold to greater than 487-fold.

### 2.5. Mouse Neutralization Assays

For in vivo assays, we first confirmed the need for a multi-antibody combination to achieve high-potency neutralization using BoNT/C and BoNT/C mAbs 1C1.1, 4C2 and 4C10. Each individual mAb, mAb pair, and the three mAb combination of 1C1.1:4C2:4C10 were evaluated in the mouse neutralization assay, where 50 µg of each mAb or an equimolar mixture of each mAb combination was mixed with BoNT/C and injected intraperitoneally in mice, and the number of mice that survived at 5 days was determined. Survival endpoints are where 50% or more of the mice survive challenge. As observed for other BoNT serotypes [31,32,52], individual mAbs were not very potent, with survival endpoints of 60–100% at 20–200 LD_50_s (Table 4). The use of mAb pairs increased potency: there were survival endpoints of 100% at 500 LD_50_ with 1C1:4C10, 70% at 2500 LD_50_ with 4C2:4C10, and 70% at 5000 LD_50_ with 1C1:4C2. Adding a third mAb further increased potency. The antibody combination 1C1:4C2:4C10 completely protected mice challenged with 20,000 mouse LD_50_s. Thus, this three mAb combination was 100 times more potent than the most potent single mAb (1C1.1).

While the above experiments illustrated the need for multiple antibodies to achieve high potency, the experiments used mAbs that were specifically chosen for their affinity for BoNT/C (190 pM-1.1 nM), rather than their cross-reactive potential. Therefore, we next evaluated the relative potencies of three mAb combinations using cross-reactive mAbs binding BoNT/C, BoNT/CD, BoNT/DC, and BoNT/D (4C4, 4C10 and 8DC4) with and without the addition of mAb 4C2. Initial experiments using BoNT/C as the challenge toxin showed the triple antibody combination of 4C4.2:8DC4.1:4C10.2 to be less effective than the combination of 1C1:4C2:4C10, with little or no survival seen against 5000 LD_50_. The addition of the mAb 4C2, which binds both BoNT/C and BoNT/DC, increased protection, with 9/10 mice surviving challenge with 20,000 LD_50_. The reason for the relative lower potency for the combination of three cross-reactive mAbs compared to the mAb combination of 1C1.1:4C2:4C10 is not known. The affinities of these mAbs are not dramatically different, so it is possible that this is due to differences between mAbs in the ability of the mAbs to bind BoNT simultaneously.

Given the increased potency of the four-mAb combination compared to the three-mAb combination versus BoNT/C, we evaluated decreasing doses of the affinity-matured antibody combination of 4C2:4C4.4:4C10.5:8DC4.4 against 40,000 LD_50_ challenges using BoNT/C, BoNT/CD, and BoNT/DC, and 10,000 LD_50_ using BoNT/D. Complete protection was seen versus BoNT/C, BoNT/CD, and BoNT/DC when 50 µg of total antibody doses were administered. Partial or complete survival against each of these three toxins was seen with antibody doses of 5.0–10 µg (Table 5). This protective efficacy is comparable to that observed for mAb combinations with other serotypes [31,32,43,53]. However, the potency of this mAb combination was somewhat lower for BoNT/D, where 80% protection was seen using 50 µg of antibody versus 10,000 LD_50_ toxin.

## 3. Discussion

The screening of human antibody diversity libraries using yeast display followed by molecular evolution to increase cross-reactivity and affinity allowed for the development of three mAbs that bound BoNT/C and BoNT/D and the mosaic toxins BoNT/CD and BoNT/DC with high affinity (K_D_ of 1.1–157 pM). The addition of a fourth mAb resulted in highly effective protection against each of these four toxins. Generally, there is little or no cross-affinity or cross-protection between different serotypes. In prior work generating more than 100 mAbs to BoNT/A, B, E, and F, only one mAb bound more than one serotype [31,32,43,52,54]. However, BoNT/C and BoNT/D proteins share a higher level of identity than most serotypes (51%), with a particularly high level of identity in the BoNT H_N_ (68%). This is a greater than average cross-serotype identity, which provided a hypothesis that the minimal number of mAbs needed to effectively protect against BoNT/C, BoNT/D, and the mosaic BoNT/CD and BoNT/D toxins might be lower than three per serotype. As part of this study, we identified two high-affinity cross-reactive mAbs that recognized the H_N_ domain, and a third high-affinity cross-reactive mAb was identified that recognized a conserved region of the LC. The addition of a fourth mAb completed a quadruple antibody combination that was able to completely neutralize 40,000 LD_50_ of BoNT/C, BoNT/CD, or BoNT/DC using 5-10 µg antibody doses. This result is comparable to what we observed with triple mAb combinations binding multiple BoNT/A [43], BoNT/E [32] and BoNT/F [31] subtypes. While the neutralization potency was lower versus BoNT/D, where the protection endpoint was at 10,000 LD_50_ using 50 µg of total antibody, considering the lower toxicity [55,56] and yields of BoNT/D compared to other toxin types, this represents a substantial protective level. The ability to effectively protect against two BoNT serotypes using only four mAbs represents significant savings in manufacturing costs and bioanalytical complexity. 

While we did not formally titer the potency of the four-mAb combination in International Units (IUs), the mouse neutralization assay data indicate that this mAb combination could theoretically yield a neutralization capacity of up to ~4 million mouse LD_50_/mg of mAb combination, which would be the equivalent of 400 IU/mg for BoNT/C, BoNT/CD and BoNT/DC, and 40 IU for BoNT/D. The lower protection against BoNT/D may reflect the lower affinity of mAb 4C10.2 for BoNT/D.

In the case of BoNT/A, the measured IU potency of a BoNT/A mAb combination was tightly correlated with the calculated neutralization capacity [43]. According to the package insert, the potency of the current therapeutic botulinum antitoxin (BAT) [41] is 3000 IU for BoNT/C and 600 IU for BoNT/D, so an equivalent mAb combination dose would be ~7.5 mg and 15 mg, respectively. In contrast, the adult dose of heptavalent equine BAT contains between 600 to 1500 mg of equine protein [41]. While the relative amounts of BAT directed against each of the seven serotypes is unknown, assuming that there is an equal amount for each serotype would give a potency for BoNT/C of 32 IU/mg and 6 IU/mg for BoNT/D, making the mAb-based antitoxins 12 and 7 times more potent for BoNT/C and BoNT/D, respectively. Moreover, the much longer half-life of human IgG mAbs to BoNT/C and BoNT/D (10–24 days) [56] compared to BAT (30 and 7.5 h, respectively) [41] eliminates the risk of rein toxication due to antibody clearance [42].

## 4. Conclusions

A human four-mAb combination was developed which neutralizes BoNT/C, BoNT/CD and BoNT/DC and BoNT/D more potently that BAT. Equine BAT is immunogenic and hypersensitivity reactions have been reported, including cardiac arrest and serum sickness [41], while fully human recombinant mAbs are significantly safer [41,57]. Other advantages of the mAb combination compared to BAT include the much longer serum half-life and the ability to make additional lots from existing cell lines without the need to start from scratch with horse immunizations.

As BoNT/C and D primarily affects animals, the human antibodies described here could be used to treat botulism in animals, with the caveat that they would potentially cause immunogenicity in non-human species and that the cost of these antibodies may be relatively high for a veterinary product considering the dosing that would be needed.

The results of a Phase 1 clinical trial evaluating a combination human/ humanized antibody product to treat botulism due to serotype A [58] support the safety of recombinant mAb combination products. The four BoNT/ C and BoNT/D mAb combination described here is highly potent and by using antibody engineering, we were able to construct a product that neutralizes two different serotypes and their mosaic toxins where a six-mAb combination would be anticipated based on prior results (three for BoNT/C and three for BoNT/D). Component mAbs were selected based on their ability to bind and neutralize BoNT/C, BoNT/D, and their mosaics. The work described here was the basis for developing the four-antibody combination drug product to treat human botulism due to BoNT/C, BoNT/CD, BoNT/DC or BoNT/D (NTM-1634) that completed a Phase 1 clinical in 2019 [56] with no drug-related serious adverse events. 

## 5. Materials and Methods

### 5.1. Ethics

The USAMRIID Institutional Animal Care and Use Committee (IACUC) approved the animal care and use protocols to conduct the animal studies reported here. Research was conducted in compliance with the Animal Welfare Act, PHS Policy, and other federal statutes and regulations relating to animals and experiments involving animals. The facility where this research was conducted is accredited by the Association for Assessment and Accreditation of Laboratory Animal Care, International (AAALAC/I) and adheres to principles stated in the Guide for the Care and Use of Laboratory Animals, National Research Council, 2011. The specific national regulations and guidelines to which this animal care and use protocol adheres are the following: (1) 7 USC, Sections 2131–2159, Chapter 54 “Animal Welfare Act”, and (CFR, Chapter 1, Subchapter A, Parts 1–4 “Animal Welfare Regulations”; (2) Health Research Extension Act of 1985, Public Law 99-158 “Animals in Research” and the Public Health Service Policy in Humane Care and Use of Laboratory Animals; (3) Biosafety in Microbiological and Biomedical Laboratories, 5th Edition, NIH, Human and Health Services Publication 21-112; (4) Army Regulation 40-33 “The Care and Use of Animals in DOD Research, Development, Test and Evaluation or Training Programs”; and (5) DOD Instruction 3216.01 “Use of Animals in DOD Programs”. DOD uses “The Guide for the Care and Use of Laboratory Animals”, 8th Edition, Institute for Laboratory Animal Research, National Research Council, as a guideline for the evaluation and accreditation of a program and it is based on the actual national regulations and guidelines for animal care and use programs. The animals used in this study were euthanized using carbon dioxide gas following the AVMA Guidelines on Euthanasia prior to spleen removal.

The University of California, San Francisco (UCSF) Institutional Review Board approved the human use protocol used for the studies described here. Human donors were laboratory workers being immunized to work with BoNT who were recruited via an informational letter and who signed an informed consent form.

### 5.2. Strains, Media, Antibodies, and Toxin

YPD medium was used for growth of *Saccharomyces cerevisiae* strain EBY100, SD-CAA, for the selection of pYD2-transformed EBY100 and SG-CAA, and for the induction of scFv expression on the surface of EBY100. *Escherichia coli* strain DH5α was used for the subcloning and preparation of plasmid DNA. 

All IgGs were expressed from Chinese hamster ovary (CHO) cells, while the mouse anti-SV5 antibody was purified from hybridoma cells and labeled with an AlexaFluor-488 (anti-SV5-AF-488) or AlexaFluor-647 (anti-SV5-AF-647) labeling kit (Invitrogen, Carlsbad, CA, USA). The secondary antibody, PE-conjugated goat anti human-Fc, F(ab) was purchased (Jackson ImmunoResearch Laboratories, West Grove, PA, USA).

BoNT/C and BoNT/DC, as pure holotoxin or toxin complexes, were obtained from Metabiologics (sold as BoNT/D but confirmed to be BoNT/DC [47]); BoNT/CD was obtained as a crude extract complex from USAMRIID, and BoNT/D was obtained as a pure complex from USAMRIID. The determination of the IU potency requires the use of a WHO-standardized antitoxin. We were not able to access such an antitoxin, and thus were unable to determine the IU using the standard assay. To calculate the IU potency from the mouse neutralization data (MNA), an ED_50_ was interpolated from the MNA data as the mAb amount at which 50% of mice survived challenge with that dose of BoNT. This ED_50_ was normalized for a 1 mg amount of mAb and divided by 10,000 mouse LD50s/IU to determine the IU potency. We have previously shown for BoNT/A that such a calculation correlates closely to the IU measured using the reference WHO antitoxin. 

### 5.3. Yeast-Displayed Human scFv Library Construction and Library Sorting

To generate human mAbs that bind BoNT/C and /D, total RNA was isolated from the blood of healthy human donors immunized with investigational pentavalent BoNT toxoid (formalin inactivated BoNT/A, BoNT/B, BoNT/C, BoNT/D, and BoNT/E). cDNA synthesis, VH, and Vk gene repertoire preparation and library construction were performed as described previously [52].

Yeast cells were grown in SD-CAA medium and the display of scFv was induced by culturing in SG-CAA with 10% SD-CAA for 48 h. For sorting, the libraries were incubated with 50 nM of BoNT/C or BoNT/DC labeled with Alexa-647 plus SV5-AF-488 at room temperature for 2 h. All subsequent washing and staining steps were performed at 4 °C using ice-cold FACS buffer (phosphate-buffered saline, 0.5% bovine serum albumin, pH 7.4). After washing, yeast clones were flow-sorted on a FACSAria II, and the population with BoNT/C binding was gated and collected. The collected yeast clones were cultured and induced for the next round of sorting. After three rounds of sorting, the collected yeast clones were plated on SD-CAA medium and cultured at 30 °C for 48 h. Binding of individual colonies was confirmed for binding using BoNT/C at progressively reduced concentrations. Unique BoNT/C binding clones were identified by DNA sequencing and best binders were converted into IgGs. Cross-reactive antibodies were isolated using BoNT/DC sorting.

For the isolation of additional BoNT/C and BoNT/DC specific scFvs, the V-genes of scFvs isolated as described above (mAbs 1C1, 4C2, 4C4, 4C5, 4C10 and 87C78) were converted to IgGs, purified, and IgG labeled with Alexa Fluor (AF)-647. The sorting protocol was then modified by incubation with unlabeled BoNT/C or unlabeled BoNT/DC (50 nM) followed by washing and then incubation with an AF-647-labeled toxin-specific IgG (2 µg/mL) plus anti-SV5-488. The use of labeled IgGs versus labeled BoNT provided a more robust staining and sorting reagent; the use of the cross-reactive IgGs allowed us to sort and analyze binding to all four toxins included in this work. Newly isolated scFvs (8DC1, 8DC2 and 8DC3) were also converted into IgGs. 

### 5.4. Measurement of K_D_ Values of Yeast-Displayed scFv

The K_D_ values of yeast-displayed scFvs were measured by flow cytometry, as previously described, with modification [54]. Briefly, 1 × 10^6^ yeast-displaying scFvs were incubated for 1 h at room temperature in FACS buffer with six different concentrations of BoNT/C BoNT/CD, BoNT/D or BoNT/DC purified holotoxins, or crude extracts, that spanned the range 10-fold above and 10-fold below the expected K_D_. Ice-cold FACS buffer was used to wash the samples, and 2 µg/mL each of an AF-647-labeled IgG that bound a different epitope was added plus anti-SV5-AF-488 at 4 °C and incubated for 60 min. Finally, the yeast clones were washed with ice-cold FACS buffer and the mean fluorescence intensity (MFl) of binding was measured by flow cytometry, as described previously [52,57].

### 5.5. Epitope Overlap Analysis

Epitopes were classified based on mAb binding competition to BoNT/C or BoNT/DC, as previously described [52,54]. Briefly, yeast-displayed scFvs were incubated for 60 min with 25 nM of one of the toxins. The ability of other IgGs to bind to the same toxin captured by the yeast-displayed scFvs was detected by incubation for 60 min with 2 µg/mL of Alexa dye APC conjugated IgG and 1 µg/mL of AF-488-labeled SV5 antibody (SV5-AF-488). The ability of the IgG to bind the scFv-captured BoNT/C or BoNT/DC, was determined by flow cytometry. The IgG that bound an overlapping epitope to yeast-displayed scFvs showed no APC signal, while those binding non-overlapping epitopes showed a positive APC signal.

### 5.6. Affinity Maturation

To increase the affinity of mAbs 1C1, 4C4, 4C10, 8DC1 and 8DC4, individual VL chain-shuffled scFv libraries were created. For each scFv library, VK gene repertoires from human immune scFv libraries were amplified by PCR using *Pfu* polymerase (Stratagene Bellingham, WA, USA) and the primers (5′-GGCGGAGGTGGCTCTGGCGGTGGCGGGTCG-3′) and PYDR1(GGTGATGGTGATGATGACCGGTACGCGTAG). To further increase VL diversity, the VL repertoire from a large non-immune scFv phage antibody library transferred from the phagemid vector pHEN1 and cloned into pYD2 was also used [59]. The VH DNA for each of the scFvs to mature was amplified from the scFv gene in pYD2 using primer PYD-F1 (5′-CCCCTCAACAACTAGCAAAGGCAGCCC-3′), that anneals upstream of the VH gene, and primer (5′-CGACCCGCCACCGCCAGAGCCACCTCCGCC-3′) that anneals in the linker gene between the VH and VL genes. The gel-purified VH gene was mixed with the gel-purified VL repertoires and combined with NcoI- and NotI-digested pYD2 vector DNA. This mixture was used to transform LiAc-treated EBY100 cells by three-fragment gap repair. Library sizes were approximately 10^7^. To select higher affinity scFvs, the VL-shuffled library was sorted as described above.

To create complementarity determining region (CDR) libraries, spiked primers were used to introduce mutations into four or five residues, located in the H1 or H3 loops of each scFv to mature, as described previously [51]. Spiked oligonucleotides were designed to have a bias for a 25% or 50% wild-type amino acid at each position, according to the codon usage. Library sizes were approximately 10^8^. 

To select higher affinity scFvs and maintain cross-reactivity, the VL-shuffled library was sorted as described above for each scFv. To maintain cross-reactivity, sorting was alternated between BoNT/C and BoNT/DC. The K_D_ values of libraries for both toxins were measured after each round to decide which toxin to use for the following sort. Alternatively, we used a dual staining protocol using recombinant BoNT/C LC-HN plus BoNT/DC and detected each with a different label antibody, e.g., incubating after toxin staining with 1C1 Alexa Fluor-488 (binds to the BoNT/C LC-HN domain only) and 4C2 AF-647 (Binds the HC domain so only BoNT/DC would be detected) plus SV5 antibody (Mouse IgG2a) and goat anti-mouse specific-PE labeled antibody (Jackson Immunoresearch #115-116-146) was used to detect expression.

### 5.7. Germline Fitting

The closest predecessor germlines for each scFv were identified using IMGT/V-Quest [60]. Sequences were compared to find the divergent amino acids. Then, mutations were restored to its germline amino acid by the QuikChange II-E Site-Directed Mutagenesis Kit (Agilent, Santa Clara, CA, USA), as described above for epitope mapping. The K_D_ values for each construct were determined as yeast-displayed scFvs using flow cytometry. Changes that did not reduce binding affinity were incorporated. After scFv gene optimization, the final clone was used as a template to continue with the next step of affinity maturation.

### 5.8. Measurement of Solution Phase Affinity at Equilibrium

For selected IgGs, the solution phase affinity at equilibrium and binding kinetics were measured using flow fluorimetry in a KinExA as previously described [54,57], except that pure BoNT/C or /DC toxins or crude culture supernatants of BoNT/CD or BoNT/D were used.

### 5.9. Measurement of In Vivo Toxin Neutralization

The mouse neutralization assay was performed as described previously [43] using female CD-1 mice. Briefly, an equimolar mixture of one to four IgG antibodies (0.5 to 50 µg total antibody) were premixed with a range of mouse LD_50_ of BoNT/C, BoNT/CD, BoNT/DC, or BoNT/D in a total volume of 0.5 mL of gelatin phosphate buffer and incubated at room temperature for 30 min. The mixture was then injected intraperitoneally into cohorts of ten mice. Due to toxin scarcity, studies using BoNT/D involved cohorts of five mice. The animals were observed multiple times daily for clinical signs of botulism. Moribund animals were euthanized. Surviving mice at the study endpoint (5 days) were tabulated. 

## Figures and Tables

**Table 1:** Percentage of amino acid identities among domains of BoNT C and D and their mosaic BoNTs.

	Holotoxin	LC	H_N_	H_C_
**BoNT/C vs.:**				
BoNT/CD	76.0%	96.2%	93.0%	41.6%
BoNT/DC	64.7%	47.1%	67.7%	75.8%
BoNT/D	51.2%	46.8%	67.9%	38.9%
**BoNT/CD vs.:**				
BoNT/DC	51.9%	47.5%	69.4%	39.1%
BoNT/D	68.7%	47.3%	69.2%	89.8%
**BoNT/DC vs.:**				
BoNT/D	76.5%	98.2%	95.8%	40.9%

LC = the light chain, or enzymatic domain; H_N_ = the translocation domain, within the amino terminal half of the heavy chain; H_C_ = the carboxy terminal half of the heavy chain, containing the receptor-binding domain. Sequences used were as described in [46].

**Table 2:** Characteristics of lead yeast-displayed scFv BoNT/C and BoNT/DC antibodies.

Clone ^a^	Epitope	Yeast-Displayed scFv K_D_ by FACS (×10^−9^ M^−1^)				IgG K_D_ (×10^−12^ M)	
		C	CD	DC	D	C	DC
4C4	H_N_	3.0	+	10.5	+	888	597
4C10	LC	1.1	+	107	+	401	ND
8DC1	H_N_	11	+	5.2	+	1800	95
8DC4	H_N_	3.0	+	39	+	1400 ^b^	591 ^b^
1C1	H_N_	0.5	NB	NB	NB	1.6	NB
1C2	ND	24	NB	NB	NB		
1C8	ND	42	NB	>200	NB		
87C1	H_N_	2.0	+	NB	NB		
87C2	H_N_	7.0	NB	NB	NB		
87C78	LC-H_N_	1.0	+	NB	NB	0.42	NB
4C1	LC	2.7	+	NB	NB		
4C2	H_C_	0.19	NB	0.5	NB	14.7	0.51
4C3	LC	4.7	+	NB	NB		
4C5	LC	0.14	+	NB	NB		
4C7	ND	64	NB	NB	NB		
4C8	ND	51	NB	NB	NB		
4C9	LC	30	+	NB	NB		
8DC2	H_C_	0.2	NB	0.5	NB	15.7	7
8DC3	H_C_	NB	NB	16.5	+	256,000	227
8DC5	LC	NB	NB	15	+		
8DC6	LC	NB	NB	43	+		
8DC7	H_C_	NB	NB	16	+		
8DC8	H_C_	NB	NB	7.3	+		
8DC9	ND	NB	NB	7.6	+		
8DC10	LC	NB	NB	12	+		
8DC11	H_C_	NB	NB	20	+		
8DC12	LC	NB	NB	73	+		
8DC13	LC	NB	NB	182	+		

The absence of binding is indicated by a “+” for studies using crude culture supernatants where the concentration of BoNT CD or BoNT/D was unknown and a K_D_ could not be calculated. NB = no detectable binding; ND = not determined. ^a^ mAbs with “C” in their name derived from sorting with BoNT/C and scFv with “DC” in their name derived from sorting with BoNT/DC. ^b^ IgG K_D_ was measured on 8DC4.1, an affinity matured version of 8DC4 resulting from light chain shuffling.

**Table 3:** Affinities of lead and final BoNT/C and BoNT/D IgG mAbs. The lead antibody appears first in the table and the final affinity matured mAb is shown below.

	Dissociation Constant, K_D_ (×10^−12^ M^−1^)			
Antibody	BoNT/C	BoNT/CD	BoNT/DC	Binds BoNT/D
**4C4**	888	ND	597	ND
**4C4.1**	570	1400	58	898
**4C4.2**	35	252	126	254
**4C4.4**	16	16	87	34
**4C10**	401	ND	107,000 (as scFv)	ND
**4C10.1**	95	374	7300	6000
**4C10.2**	34	0.73	892	1450
**4C10.5**	1.1	2.43	15	17
**8DC1**	1809	ND	95	ND
**8DC1.2**	779	426	174	370
**8DC4**	3000 (as scFv)	ND	39,000 (as scFv)	ND
**8DC4.1**	1400	591	1400	1400
**8DC4.4**	136	117	146	157
**8DC4.5**	147	38	25	12

ND = not determined.

**Table 4:** Protection of mice against intraperitoneal challenge with BoNT/C.

LD_50_ of BoNT/ C	20	200	500	2500	5000	10,000	20,000	40,000
**mAbs**	Mice surviving/10 mice treated							
1C1.1	10/10	10/10	1/10					
4C2	6/10	0/10						
4C10	6/10	0/10						
1C1.1:4C2			10/10	10/10	7/10	2/10		
1C1.1:4C10			10/10	3/10	2/10			
4C2:4C10			10/10	7/10	2/10			
1C1.1:4C2:4C10						10/10	10/10	1/10
4C4.2:8DC4.1:4C10.2					2/10	0/10		
4C2:4C4.2:8DC4.1:4C10.2							9/10	0/10

The indicated amount of BoNT and 50 µg of the indicated single mAb or an equimolar combination of the indicated two or three mAbs combinations were injected intraperitoneally (I.P.) into cohorts of ten mice and the number of mice surviving was determined.

**Table 5:** Endpoint protection of the four-antibody combination 4C2:4C4.4:4C10.5:8DC4.4 versus 40,000 LD_50_ BoNT/C, BoNT/CD and BoNT/DC and 10,000 LD_50_ BoNT/D.

	Number of Survivors/Total Mice Treated			
Toxin Used and Lot	BoNT/C C112913-01	BoNT/CD CDU 021113	BoNT/DC D010604-01	BoNT/D U 021113
**Total Antibody Dose per Mouse**				
50 µg	10/10	10/10	10/10	4/5
10 µg	10/10	5/10	10/10	
5.0 µg	10/10	5/10	0/10	
1.0 µg	0/10	0/10	0/10	
0.5 µg	0/10	0/10	0/10	

## Data Availability

We have not disclosed the sequences of the antibodies described here because their intended use is for biodefense. All other data is included in this manuscript.
